# Effect of Magnesium Powder Application on the Microstructure and Properties of Rods Extruded by the Forward-Backward Rotating Die Extrusion Method

**DOI:** 10.3390/ma15124094

**Published:** 2022-06-09

**Authors:** Anita Olszówka-Myalska, Patryk Wrześniowski, Paweł Ostachowski, Marcin Godzierz, Dariusz Kuc

**Affiliations:** 1 Faculty of Materials Engineering, Silesian University of Technology, Krasińskiego 8, 40-019 Katowice, Poland; patryk.wrzesniowski@polsl.pl (P.W.); dariusz.kuc@polsl.pl (D.K.); 2 Faculty of Non-Ferrous Metals, AGH University of Science and Technology, Al. A. Mickiewicza 30, 30-059 Kraków, Poland; pawel.ostachowski@agh.edu.pl; 3 Centre of Polymer and Carbon Materials, Polish Academy of Sciences, M. Curie-Skłodowskiej 34, 41-819 Zabrze, Poland; mgodzierz@cmpw-pan.edu.pl

**Keywords:** magnesium-based materials, powder metallurgy, extrusion, SPD, KOBO, cellular structure

## Abstract

The effects of severe plastic deformation (SPD) with a forward-backward rotating die (KOBO extrusion) on pure magnesium, in the form of cold-compacted powder, sintered powder, or cast ingots as reference, were examined. This method is known to reinforce metals, but the role of the initial form of magnesium applied in the fabrication of metal-based rods, as well as related phenomena, has not been characterized until now. The problem is important in the potential processing of commercial metal powders, the recycling of metal shavings, and the fabrication of metal matrix composites with discontinuous reinforcing phases. In the presented experiments, rods of 8 mm in diameter and 400 mm in length were obtained, and the structural effects induced by KOBO that occurred on a macro- and microscale on the surface and cross sections were characterized. Changes in the size and orientation of α-Mg crystallites were determined by XRD. The porosity, hardness, tensile strength, and compressive strength were measured, and the mechanisms of decohesion dependent on starting metal form were analyzed. After KOBO extrusion, significant differences were observed in the microstructure and properties between the materials derived from cold-compacted powder, sintered powder, and reference cast magnesium. Due to the application of KOBO, apart from α-Mg grain refinement, the MgO derived from the initial powder’s surface was refined to fine regular particles surrounded by magnesium. Their bands curved in the perpendicular plane and were oriented with the extrusion direction of the formed network, which augmented some mechanical properties and changed the decohesion mechanism. The conducted experiments revealed that before extrusion by KOBO, the magnesium powder required sintering under pressure.

## 1. Introduction

Various technologies can be used for the fabrication of metal items, but casting and powder metallurgy are the leading techniques. In addition, the manufacture of metal rods, wires, or sheets needs many forming and heat-treating techniques. Usually, for this type of product, cast ingots of various shapes and sizes are used as raw materials, but in some cases metal powders or shavings are used instead [1,2,3,4,5,6,7,8,9]. The processing of metal matrix composites, apart from appropriate metal deformation, additionally requires a strong bonding between the components and a homogenous distribution of the reinforcing phases (particles or discontinuous fibers) [10,11,12,13,14]. In this case, to obtain metal matrix composite rods or another composite product of suitable geometry, ingots fabricated by stir casting or powder component mixtures are usually employed.

The issue considered in this study is the application of severe plastic deformation (SPD) with a forward-backward rotating die (KOBO) [15,16,17,18,19,20,21] using magnesium powder as a raw material. This type of extrusion belongs to the large group of SPD methods extensively described in the literature for the processing of various materials, but in contrast to previously described methods also enables the fabrication of rods with cross sections of various shapes and sizes. The SPD methods that are most frequently used and described in the literature are: equal channel angular extrusion [22,23,24], high-pressure torsion [25,26,27], accumulative roll bonding [28,29,30], repetitive corrugation and straightening [31,32,33], and asymmetric rolling [34,35,36]. The aim of all of them is the intense refining of the metal grain size in order to obtain superior mechanical properties. The usefulness of SPD methods in the processing of metal matrix composites has also been examined, and it was found that they can ensure an effective augmentation in the mechanical properties of the composites [37,38,39,40,41]. These advantageous properties are mainly due to metal grain refinement, while the other components present largely play the role of functional additions changing the tribological, electrical, or thermal properties. The lower reinforcing effect of particles or short fibers is related to phenomena induced at the metal/reinforcing phase interface by intense plastic deformation.

In the case of the KOBO method, better mechanical properties were obtained after the application of cast alloys of aluminum [17,42,43], magnesium [44,45,46,47,48,49,50], and copper [51,52,53,54,55], as well as composite ingots with matrices of aluminum [56], magnesium [57], and silver matrix composites [58]. The application of magnesium powder to KOBO extrusion is a new technological concept, which requires studies covering potential rod fabrication from magnesium powders, shavings, and matrix composites with functional phases. The implementation of these concepts requires a thorough knowledge of the structural effects occurring in magnesium powder processed by KOBO and its influence on decohesion effects.

In comparison with the extrusion of cast raw materials, an additional structural element, MgO, which arises from the presence of a surface oxide film on magnesium powders or shavings, is an inherent feature affecting powder grain consolidation, both in cold and hot forming techniques. The phenomena occurring in powder consolidation start to be even more complex when nonmetallic submicro and nanosized components, such as nanoparticles, nanotubes, and nanoflakes, are added to the magnesium [59].

In the experiments presented herein magnesium rods extruded by the KOBO method and obtained from three different raw materials—mechanically compacted magnesium powder, sintered magnesium hot pressed in a vacuum, and an ingot of pure magnesium for comparison—were characterized. The examinations were focused on structural phenomena observed both on the rods’ surfaces and their cross sections. The tensile and compressive strength were determined, and structural effects occurring at the fractures were analyzed. The results obtained allowed the most convenient initial form of magnesium powder for KOBO processing to be determined. Moreover, the results presented in this study can be helpful in understanding the phenomena occurring in magnesium matrix composites with micro- and nanosized reinforcement that are processed by SPD methods. The results of KOBO extrusion for the Mg-CNT system will be presented in a further paper.

## 2. Materials and Methods

**Figure 1:**
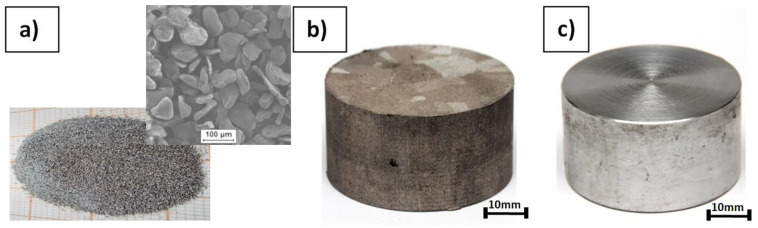
Raw materials used for metal rod processing by KOBO extrusion: (**a**) Mg powder macro and microstructure before compaction, (**b**) sintered Mg billet, (**c**) cast Mg billet.

**Figure 2:**
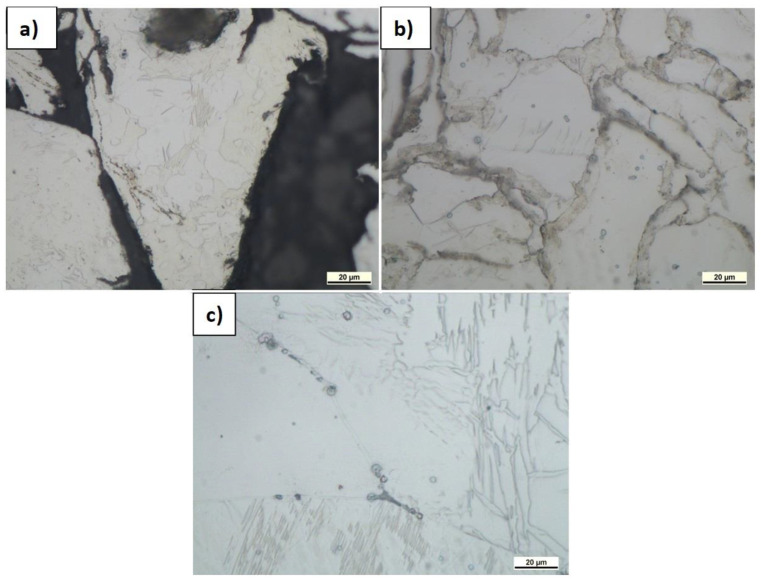
LM micrographs of raw material cross sections (etched): (**a**) Mg powder embedded in resin, (**b**) sintered Mg, (**c**) cast Mg.

Three types of raw material were chosen for rod fabrication with KOBO extrusion: pure magnesium powder (63–250 µm, product number 13112, Sigma Aldrich, Saint Louis, MO, USA) treated by cold compaction under a pressure of 30 tons (sample diameter 40 mm), sintered magnesium obtained from the same powder by hot pressing under vacuum (temp. 350–600 °C, pressure 1.5–15 MPa), and magnesium of technical purity as a reference. Figure 1 shows macrographs of the initial samples used for the experiments, while Figure 2 shows the micrographs of their etched cross sections (33 mL H_2_O, 66 mL C_2_H_5_OH, 1 mL HNO_3_, 2 g citric acid) obtained by light microscopy (LM) (Nikon Eclipse MA-200, Nikon Metrology, Tokyo, Japan).

**Figure 3:**
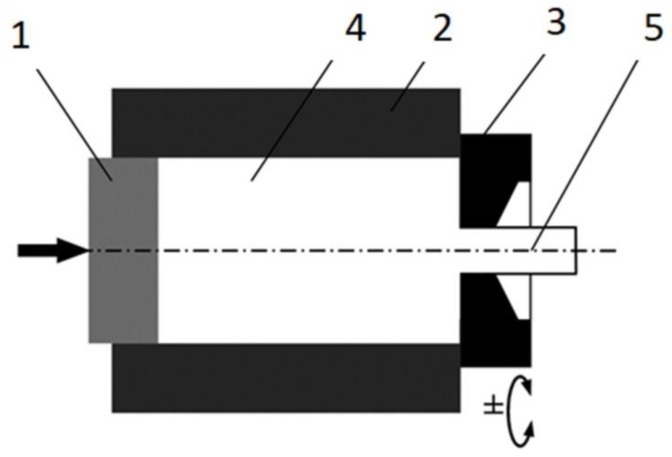
A scheme of direct extrusion by the KOBO method: 1—punch, 2—container, 3—reversibly rotated die, 4—extruded metal/billet, 5—product (rods).

**Figure 4:**
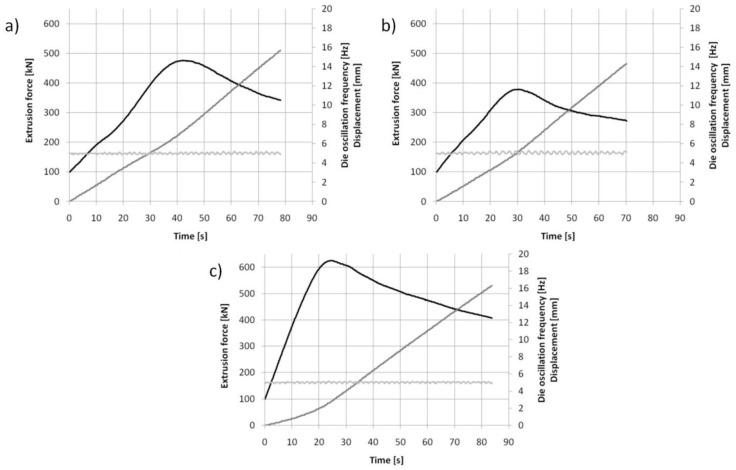
Curves of extrusion force vs. time registered for KOBO extrusion at room temperature with frequency 5 Hz, extrusion rate 0.2 mm/s, and extrusion ratio λ = 25 for: (**a**) compacted magnesium powder Mg(P), (**b**) sintered magnesium powder Mg(S), and (**c**) cast magnesium Mg(C).

KOBO extrusion was conducted using a hydraulic press with a maximum extrusion force of 1 MN. Initial samples, 40 mm in diameter and 20 mm high, were reversibly twisted with a constant angle of ±8° just before entering the die, where the cross section was reduced to 8 mm (extrusion ratio λ = 25). After obtaining the maximum force, a die oscillation frequency of 5 Hz and an extrusion rate of 0.2 mm/s were applied. Neither the samples nor any part of the equipment were additionally heated, so the process can be classed as cold extrusion. However, the processes occurring in the material and its friction with the die induced heat, and the temperature determined with a thermographic camera just behind the KOBO die was estimated at approximately 200 °C [60]. A scheme of the KOBO extrusion is shown in Figure 3. The curves of extrusion force vs. time registered for each KOBO process are presented in Figure 4, while Table 1 shows the measured maximum force, as well as the required time and displacement to reach that force. The following abbreviations are used: Mg(P)—compacted magnesium powder, Mg(S) sintered magnesium, and Mg (C)—cast magnesium.

The macrostructure of the rod surfaces and non-etched cross sections was examined, and the microstructure of the polished and etched (I step 33 mL H_2_O, 66 mL C_2_H_5_OH, 1 mL HNO_3_, 2 g citric acid, II step 100 mL C_2_H_5_OH, 10 mL HCl, 3 mL HNO_3_) samples was then characterized by LM, revealing the arrangement of both α-Mg and MgO.

X-ray diffraction (XRD) methods were employed for the characterization of crystallite size in the raw materials and KOBO-processed rods, using a D8 Advance diffractometer (Bruker AXS, Karlsruhe, Germany) with a Cu-Kα cathode (λ = 1.54 Å) operating at 40 kV voltage and 40 mA current. The scan rate was 0.60°/min, with a scanning step 0.02° in a range of 20° to 120° 2θ. The identification of fitted phases was performed using the DIFFRAC.EVA program with the ICDD PDF-2 database, while the exact lattice parameters of the fitted phases were calculated using Rietveld refinement with the TOPAS 6 program, based on Williamson-Hall theory. The pseudo-Voigt function was applied to the description of diffraction line profiles by the Rietveld refinement [61,62,63,64]. The R_wp_ (weighted-pattern factor) and S (goodness-of-fit) parameters were used as numerical criteria of the quality of the fit calculated with respect to the experimental diffraction data [63].

The open porosity of the obtained rods, the initial sinter, and the reference cast magnesium was measured by the Archimedes method (PN-EN 993-1:2019-01). Their hardness was determined by the Vickers method (HV0.2) with a Zwick 110 hardness tester (ZwickRoell, Ulm, Germany). Rods of diameter ϕ = 8 mm were then prepared by machining samples for tensile strength tests (h = 70 mm, ϕ = 8 mm; gauge h = 40 mm, ϕ = 5 mm) and also for compressive strength tests (h = 14 mm, ϕ = 7 mm). These diameter reductions allowed the macrodefects on the rod surfaces that formed in the KOBO extrusion to be removed. The mechanical property investigations were conducted for tensile strength with an MTS-Landmark 100 kN (MTS Systems, Eden Prairie, MN, USA) and an Instron 4400 (Instron, Norwood, MA, USA) for compressive strength. For mechanical properties examinations, for each case, tests were applied on 3 samples taken from the end part of extruded rods. The microstructure of the fractured surfaces formed in both tests of mechanical properties was characterized by scanning electron microscopy using a Hitachi 3400 N (Hitachi, Tokyo, Japan).

## 3. Results and Discussion

### 3.1. Macrostructure and Microstructure

**Figure 5:**
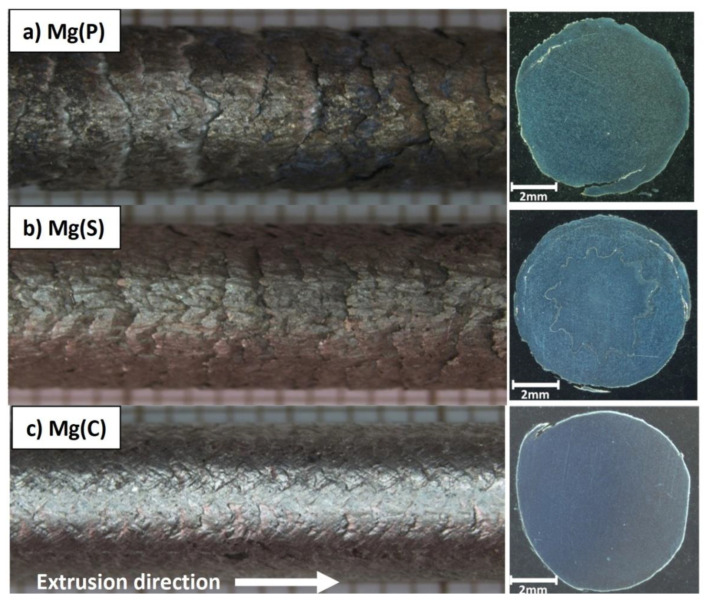
Macrographs of magnesium rod surfaces and cross sections obtained from: (**a**) compacted magnesium powder—Mg(P), (**b**) sintered under pressure magnesium powder—Mg(S), (**c**) cast magnesium—Mg(C).

The macrostructure of the magnesium rod surfaces and the polished and non-etched cross sections after KOBO extrusion are shown in Figure 5. On the shiny surfaces of both the Mg(P) and Mg(S) rods, regular plates resembling fish scales were formed, oriented in line with the extrusion direction. Moreover, the length and depth of the scales on the Mg(P) rod were higher than those on the Mg(S) rod, being approx. 1.9 mm and 1.3 mm in length and 0.7 mm and 0.4 mm in depth, respectively. On the Mg(C) rod, which was also shiny, only a regular relief in the form of crossing lines oriented at 45° to the extrusion direction was observed.

The results of the open porosity measurements are presented in Table 2, and they are in good agreement with the macroscopic observations and well-known effects usually induced by plastic working. The extrusion process caused a decrease in porosity in both the sintered and cast magnesium. In the case of rods obtained from cold compacted magnesium powder, where the scales were longer and deeper, the open porosity was the highest. Additionally, it must be mentioned that the KOBO extrusion of the compacted powder gave a porosity less than of that the sintered sample prior to extrusion (Table 2). Generally, the effect of scale formation on the rod surfaces and the differences in porosity can be explained by the high friction between the KOBO die and magnesium enriched in MgO, as well as by the interaction between single powder grains covered with MgO film. The size of the scales, both in length and depth, was several dozen percent higher for Mg(P) than for Mg(S). This effect comes from a weaker bonding between powder grains in the Mg(P) sample, since they were only mechanically compacted, meaning that the rods had greater friction with the die surface, while their movement and deformation were both easier.

The analysis of force vs. time curves for KOBO extrusion (Figure 4 and Table 1) showed that the value of maximum force necessary for rod fabrication was highest for the cast initial material, and its maximum was reached in a shorter time compared to the other two samples. This behavior suggests that plastic deformation and changes in grain size are the main processes in cast magnesium structure transformation. The comparison of the curves for cold compacted and sintered powder extrusion revealed that a higher maximum force and a longer processing time were needed for the compacted powder. In this case the movement of the raw powder grains seemed to be easier due to weak bonding, but the friction between them was more intense. The observed differences in KOBO processing (maximum force value, time, and displacement) between cold compacted powder, sintered powder, and cast magnesium indicated that the consolidation and grain refinement induced by SPD can give different effects on magnesium due to the presence of an MgO film in the raw material. This issue will be presented in greater depth later in this paper.

**Figure 6:**
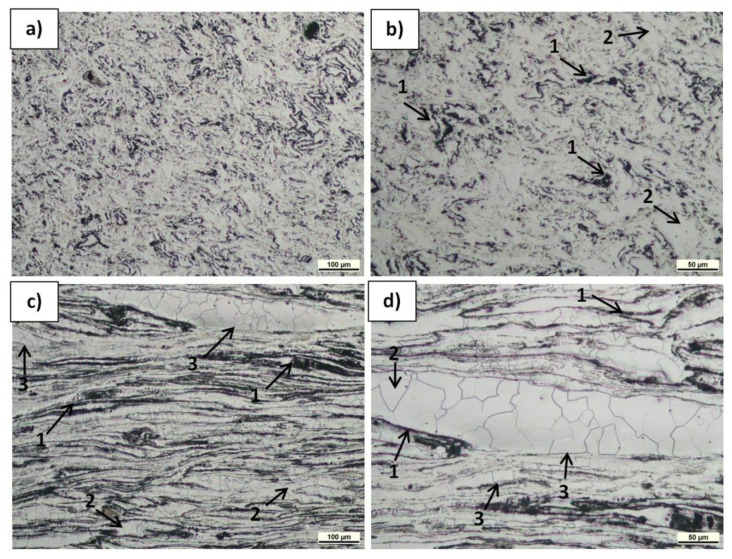
LM micrographs of etched transverse (**a,b**) and longitudinal (**c,d**) sections of Mg(P) rod KOBO-extruded: 1—MgO bands, 2—α-Mg grains, 3—α-Mg grains of shape determined by MgO bands position.

**Figure 7:**
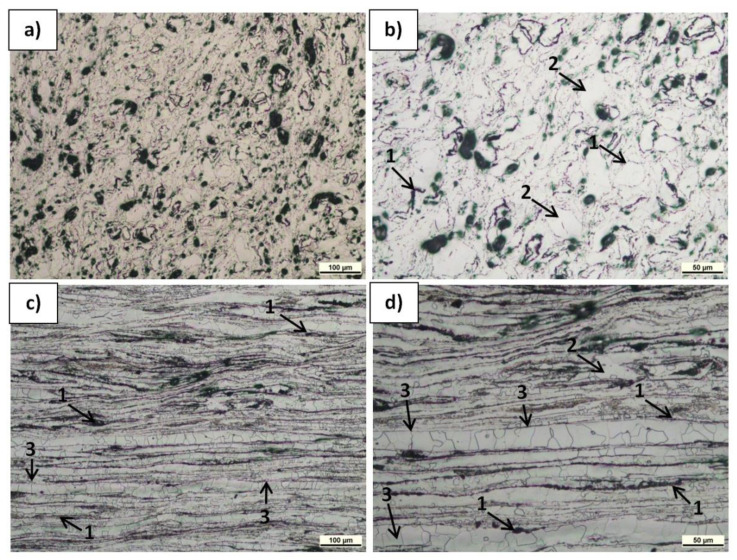
LM micrographs of etched transverse (**a,b**) and longitudinal (**c,d**) sections of Mg(S) rod KOBO-extruded: 1—MgO bands, 2—α-Mg grains, 3—α-Mg grains of shape determined by MgO bands distribution.

**Figure 8:**
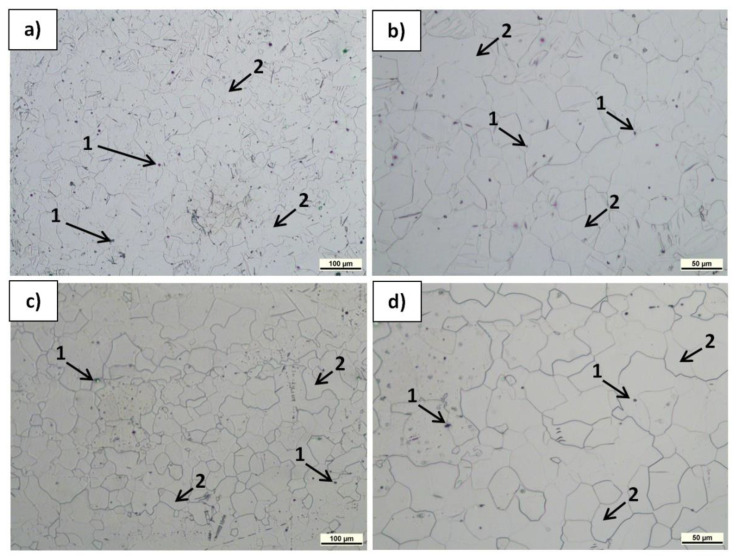
LM micrographs of etched transverse (**a,b**) and longitudinal (**c,d**) sections of Mg(C) rod KOBO-extruded: 1—single MgO particles, 2—α-Mg grains.

The microstructure of the polished and etched samples at transverse and longitudinal sections was characterized by LM, and the results are presented in Figure 6, Figure 7 and Figure 8. The observations revealed α-Mg grain boundaries and MgO (very fine spherical dark phases) in all the materials, but differences in their content and arrangement, depending on the sample type and plane orientation, could be observed. In the Mg(P) material in the section transverse to the extrusion direction (Figure 6a,b), the refined oxides formed slightly curved chains or accumulated around the α-Mg grains. In the Mg(S) rod (Figure 7a,b), both effects were also visible, but the concentration of refined oxides was less. In the Mg(C) rod (Figure 8a), only single and very fine oxides were noted. The longitudinal sections of the Mg(P) rod exhibited (Figure 6c,d) oxide bands that were also present in the Mg(S) rod (Figure 7c,d) but were thinner. In the case of the Mg(C) rod (Figure 8b), in the longitudinally oriented sample, only very fine single oxides unconnected with each other were noted.

The concentration of oxides was higher in the Mg(P) sample compared with the Mg(S) sample, but the band distances were similar. This effect could have been a consequence of the consolidation conditions for the Mg powder. Due to friction in the air atmosphere during cold compaction and subsequent KOBO extrusion, further oxidation of newly formed oxide-free microareas may have occurred.

In the LM examinations, qualitative differences in the size of the α-Mg grains and their shape induced by KOBO extrusion were noted. The size of the α-Mg grains in the Mg(P) and Mg(S) was similar and evidently less than in Mg(C). Moreover, in Mg(P) and Mg(S) some grain boundaries shape was determined by the oxide band position (Figure 6c,d and Figure 7c,d). Therefore, apart from the band orientation occurring at the longitudinal section, some α-Mg grains boundaries were also oriented. Both differences in grain size and shape can be explained by differences in the dynamic recrystallization. In the case of pure cast magnesium applied as initial material, the grain growth was not limited by oxides, while in the compacted or sintered powder the metal deformed by SPD processing was restricted by being between the oxide bands. Deeper analysis of structural changes will be conducted in the future with TEM (Transmission Electron Microscopy), EBSD (Electron Back Scattered Diffraction), and quantitative image analysis.

XRD was employed in this study, and the lattice parameters, lattice strain, and the crystallite size in the perpendicular and longitudinal orientations to the extrusion direction were determined. The results are presented in Table 3.

Rietveld refinement studies of our Mg-based materials showed the presence of just the α-Mg phase (P6_3_/mmc space group), with a minor variation in lattice parameters (Table 3). The highest lattice strain and smallest crystallite size was found in magnesium powder, which can be explained by the rapid crystallization in the fabrication process for such powders. Sintering caused a twofold increase in crystallite mean size and a simultaneous strain decrease. KOBO extruded rods (Mg(P)) fabricated from cold compacted powder exhibited a lattice strain decrease and crystallite size increase. They were oriented with the extrusion direction, and their longitudinal size was 16% higher than their perpendicular. In the case of extruded sintered material (Mg(S)), the lattice stain was the highest, but the crystallite size was less than in the initial sintered material, and they were also oriented with respect to the direction of extrusion, with their longitudinal size being 29% higher than their perpendicular. Compared to the initial sintered material, these values decreased by 42% in the perpendicular and 25% in the longitudinal orientations. Examination of the reference material showed a decrease in crystallite size of ~8%, but no orientation was detected, and this result indicated the effect of full dynamic recovery and recrystallization occurring in KOBO-processed rods produced from cast initial material.

As shown by XRD, the size of the crystallites was less for rods processed from magnesium powder than for those fabricated from cast magnesium, while anisotropy dependent on the extrusion direction was also noted for the powder-derived material. These results are in good agreement with the LM observations, and they confirm the influence of MgO bands on magnesium refining and recrystallization processes. It must be mentioned that XRD analysis of the MgO microstructure changes induced by magnesium powder KOBO processing was not possible, due to the crystalline oxide content being insufficient for that method.

### 3.2. Mechanical Properties and Decohesion Effects

The results of hardness measurements (HV0.2) presented in Table 4 exhibited evident differences, depending on the raw material applied in the KOBO extrusion. The hardness of the materials obtained from magnesium powder was slightly higher in their transverse section than in their longitudinal section, and it was evidently higher than that of the material extruded from cast magnesium, where no hardness anisotropy was detected. This effect can be explained by differences in microstructure, such as the finer α-Mg grain size in the Mg(P) and Mg(S) rods, which was visible in the LM images, and the presence of very fine MgO phases, for the influence of MgO also affected the measurements in the transverse and longitudinal sections. Two reasons are possible for the higher hardness of Mg(S) than Mg(P) rods—the slightly higher porosity of the Mg(P) sample and the higher content of MgO, which can form bands of agglomerated particles. The role of potential differences in grain size distribution needs further examination.

The results of tensile and compressive strength tests are presented in Table 4 and Figure 9, Figure 10, Figure 11, Figure 12, Figure 13, Figure 14, Figure 15 and Figure 16.

For the tensile strength examination, it was found that the UTS and 0.2% OYS, as well as the strain, gave their best values with the Mg(C) rods, but the Young’s modulus was higher for the rods obtained from powdered magnesium, both sintered, Mg(S), and cold compacted, Mg(P). Higher values of the Mg(C) UTS, 0.2% OYS and the strain, despite of higher crystallites size than other materials, can be explained by it lower porosity. The lower Young’s modulus is due to the lack of refined MgO grains. The comparison of Mg(S) and Mg(P) rods showed that all the parameters determined by the tensile strength tests were higher for the previously sintered powder-derived material.

Differences in the macro- and micro-observations of rods that were fractured in the tensile strength tests were revealed, depending on the applied raw material. In the macroscale images (Figure 11 and Figure 12), both in the Mg(P) and Mg(S) rods, the fracture occurred without neck formation and was of the cup and cone type. Additionally, in case of the broken Mg(P) specimen, two zones (Figure 11), the inner one with dimensions of approx. 3.2 mm and the outer resembling a ring approx. 0.9 mm thick, occurred at the cross section, both in the cup and cone parts of the fracture. In the SEM micrographs, bands of deformed metal were observed, oriented with the extrusion and the force applied in the tensile strength test. In the perpendicular plane they formed very characteristic bands separated by voids of a similar shape. Moreover, very fine and regular oxide phases were detected on the surface of the magnesium bands. These results indicated the formation of a 3D network by the oxide/refined α-Mg mixture, which was oriented with respect to the KOBO direction. Microareas of small dimples, typical for plastic deformation, were occasionally visible with higher magnification. The fracture morphology was quite similar for both sample zones, but that inside was less compact and the irregular slits between the deformed metal were more frequent, indicating worse compaction and powder consolidation during the extrusion. The macroscale images of fractured Mg(P) (Figure 11a,b) revealed that the outer zone was stronger and cracked in a brittle manner, while the formation of a characteristic cone suggested a shear fracture. These observations are in good agreement with effects visible on a microscale and confirm the influence of refined oxides on the fracture mechanism. The morphology of the fractured Mg(P) sample indicates the necessity of strong control of technological parameters when only a compacted powder is used as raw material.

The Mg(S) fracture (Figure 12) of a cone and cup type also revealed bands of deformed metal, consisting of very fine grains with narrow irregular cracks between them. Moreover, fine oxides strongly bonded with the metal and fine dimples confirming the plastic deformation effects were found at the surface of the bands. This type of decohesion explains the better properties of Mg(S) compared with Mg(P). Extrusion refined the magnesium grain size for both Mg(P) and Mg(S), as was revealed by microstructure studies (Figure 6 and Figure 7, Table 3), but previous powder sintering followed by extrusion ensured better material consolidation and influenced the thin film transformation processes.

The curvature and orientation observed in fractured Mg(S) rods were characteristic for deformed metal bands and were in good agreement with the previously presented LM microphotographs of materials just after extrusion. The MgO particles that were refined in the KOBO extrusion formed bands curved in the perpendicular plane (Figure 7a,b), and the similar surface morphology of the refined magnesium bands with MgO (Figure 12) indicated their influence on the rods’ decohesion and crack propagation.

**Figure 9:**
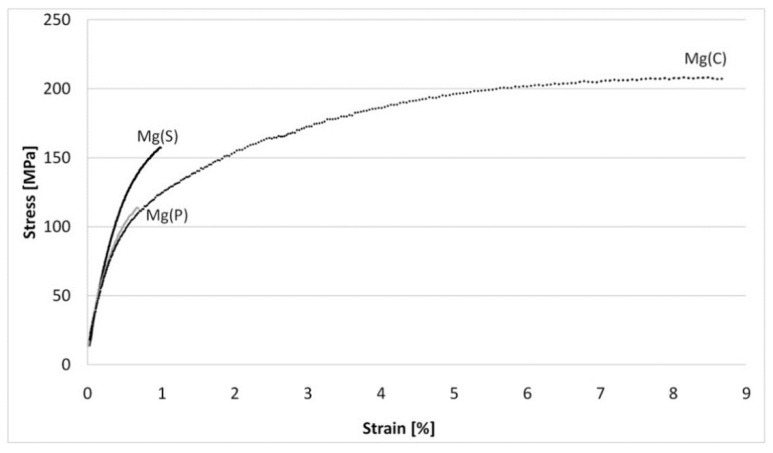
Representative curves obtained in tensile strength tests for magnesium rods extruded by the KOBO method from cold compacted magnesium powder—Mg(P), sintered magnesium powder—Mg(S), and cast magnesium—Mg(C).

Analysis of the shape of the stress-strain curves (Figure 9) indicated that for Mg(S) and Mg(P) the evident stress maximum of the curves was not obtained. This effect was probably induced by structural internal defects (porosity)and in conjunction with results from the examination of fractured samples suggested correction of the consolidation parameters, particularly for the Mg(P) rods. In the case of the reference Mg© rod, the fracture in the macroscale images also occurred without neck formation, but with an axis of 45° as in the typical brittle samples. Observations at a microscale (200×) showed that the decohesion occurred between very fine grains, while a further magnification increase (1000× and more) revealed typical but very fine dimples, suggesting plastic deformation as the dominant effect. This behavior of SPD-processed cast magnesium was previous described by other authors [65,66,67,68].

The samples taken from the Mg (C) rod subjected to the static tensile test showed a typical shear fracture (Figure 13b). The obtained slip fracture (Figure 13a,c) was the result of cracking in the planes having the highest tangential stress, which occurred at an angle of 45° to the stretching direction. The fracture surfaces usually had a brittle character, characteristic of a skid crack (Figure 13d–g). However, there were areas with characteristic craters indicating plastic flow of the α-Mg matrix metal (Figure 13h,i).

From the compressive strength examination of the rods derived from powdered raw materials (Table 4), it was found that the σ_max_, YS, and E were higher in the case of previous sintering (Mg(S) rod), which is in good agreement with the results of tensile strength tests and microhardness measurements. The comparison with the©(C) rod showed that the σ_max_ of Mg(S) was approx. 10% less, but the YS approx. 140% and the E value approx. 27% higher. Also, the Mg(P) rod exhibited evidently higher YS (85%) and E (18%) values than © Mg(C) rod.

Macro- and micro-observations of the samples fractured in compressive strength tests presented in Figure 14, Figure 15 and Figure 16 demonstrated differences induced by processing parameters. In the Mg(P) rod after the compressive test (Figure 14), fractures of a cup and cone type, resembling those in the tensile strength test, were observed, along with cracks at the machined sample surface and inside the sample, these having different orientations. The cone was compact, but the cup consisted of either compacted split metal or metal fibers, and they were separated by cracks of various sizes. This failure mechanism indicated differences in rod properties at the cross section, which were revealed earlier by the tensile strength. Additionally, oxide phases that were compact in shape and very fine (1 μm and less) were present in the metal (Figure 14e–h) in the form of both split material and fibers.

In the Mg(S) broken specimen (Figure 15), decohesion occurred with a plane oriented at 45° to the extrusion direction, and on the machined sample surface only some sparse and curved cracks were visible. The microstructure of the fractured surface contained parallel-oriented magnesium bands with a smooth surface that suggested brittle cleavage fracturing, and very fine oxide particles mixed with strongly refined magnesium (Figure 15h,i). These results explain the generally better properties in comparison with those of Mg(P) and some of those of Mg(C).

The fracture in the reference Mg(C) rod (Figure 16) occurred with a plane oriented at 45°, and at the fractured surface faults/cracks oriented perpendicularly to the extrusion and compressive force were observed independently of the applied magnification. Crack formation at the machined sample surface, as seen in the previously examined materials fabricated from the powders, was not noted. The microstructure of the fractured surface showed brittle cleavage with microareas of fine dimples visible with higher magnification.

**Figure 10:**
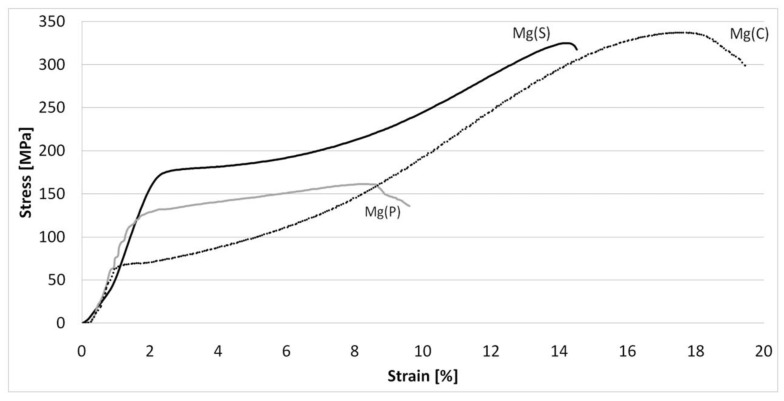
Representative curves obtained in compressive strength tests for magnesium rods extruded by the KOBO method from cold compacted magnesium powder—Mg(P), sintered magnesium powder—Mg(S), and cast magnesium Mg(C).

**Figure 11:**
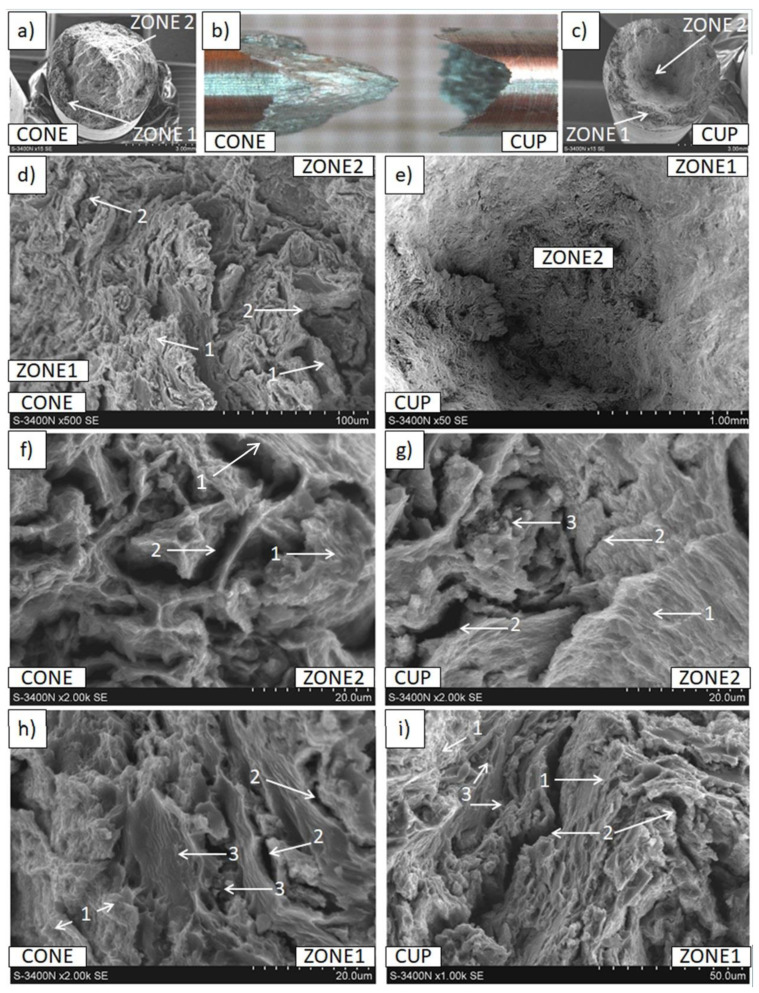
Decohesion effects in Mg(P) rod after tensile strength test. View of sample, fracture of cone and cup type with two zones inside and outside (**a**–**c**). SEM micrographs of fracture surface (**d**–**i**) with plastic deformed bands (1), cracks (2), and fine oxides (3).

**Figure 12:**
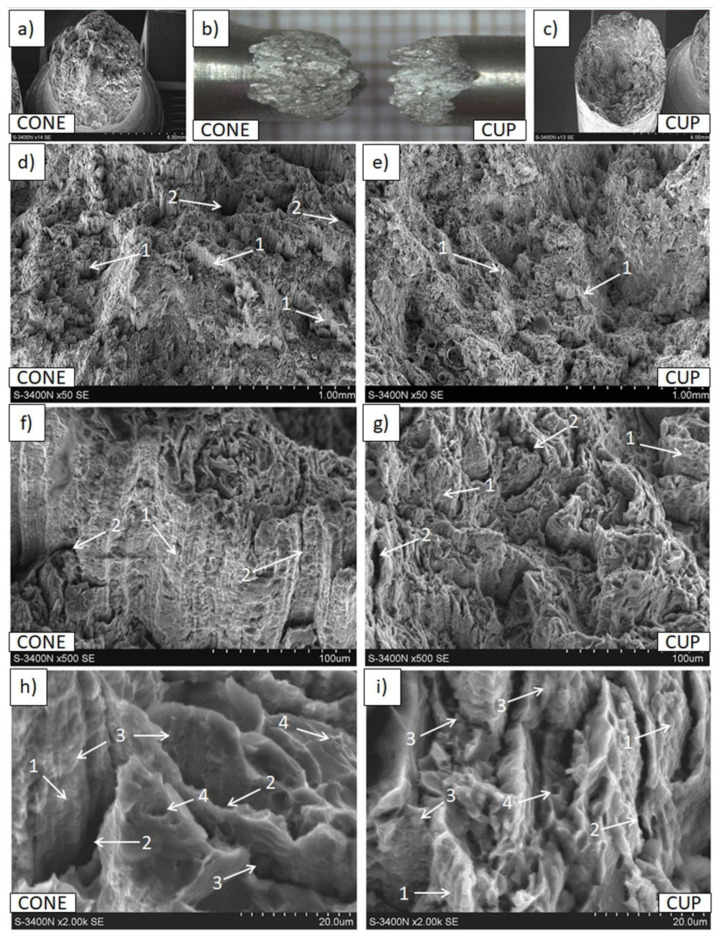
Decohesion effects in Mg(S) rod after tensile strength test. View of sample, fracture of cone and cup type (**a**–**c**). SEM micrographs of fracture surface (**d**–**i**) with plastic deformed and curved metal bands (1), cracks (2), fine oxides (3), and dimples of plastically deformed α-Mg (4).

**Figure 13:**
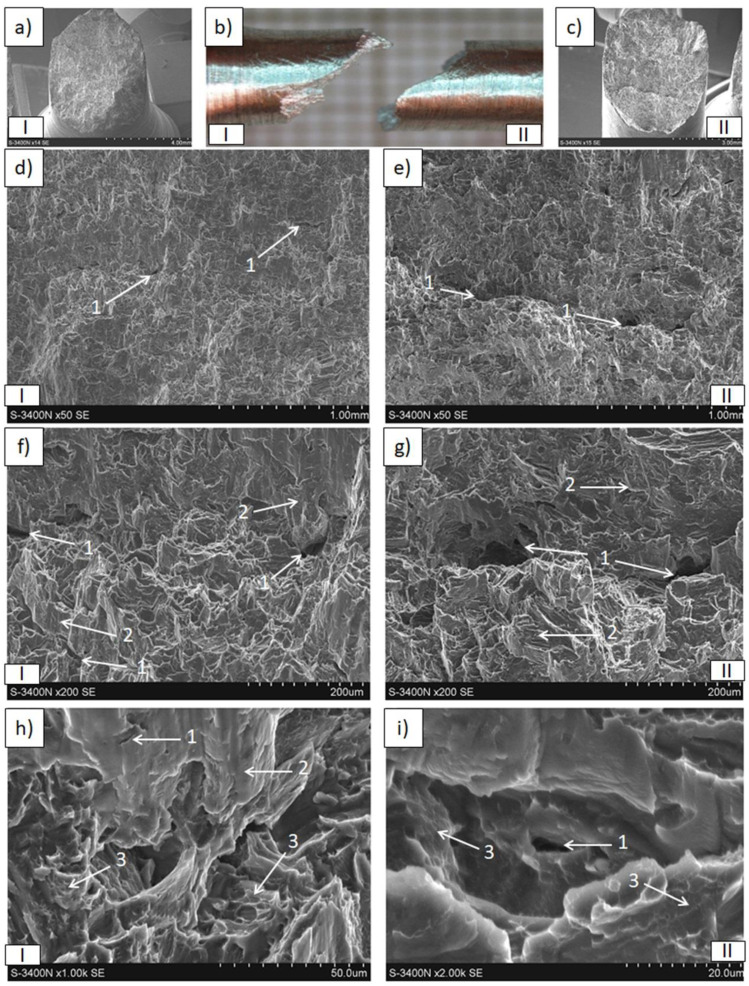
Decohesion effects in Mg(C) rod after tensile strength test. View of sample, fracture of quasi-brittle type with approx. 45° angle (**a**–**c**). SEM micrographs of fracture surface (**d**–**i**) with cracks perpendicular to extrusion direction (1), cleavage metal decohesion (2), and dimples of plastically deformed α-Mg (3).

**Figure 14:**
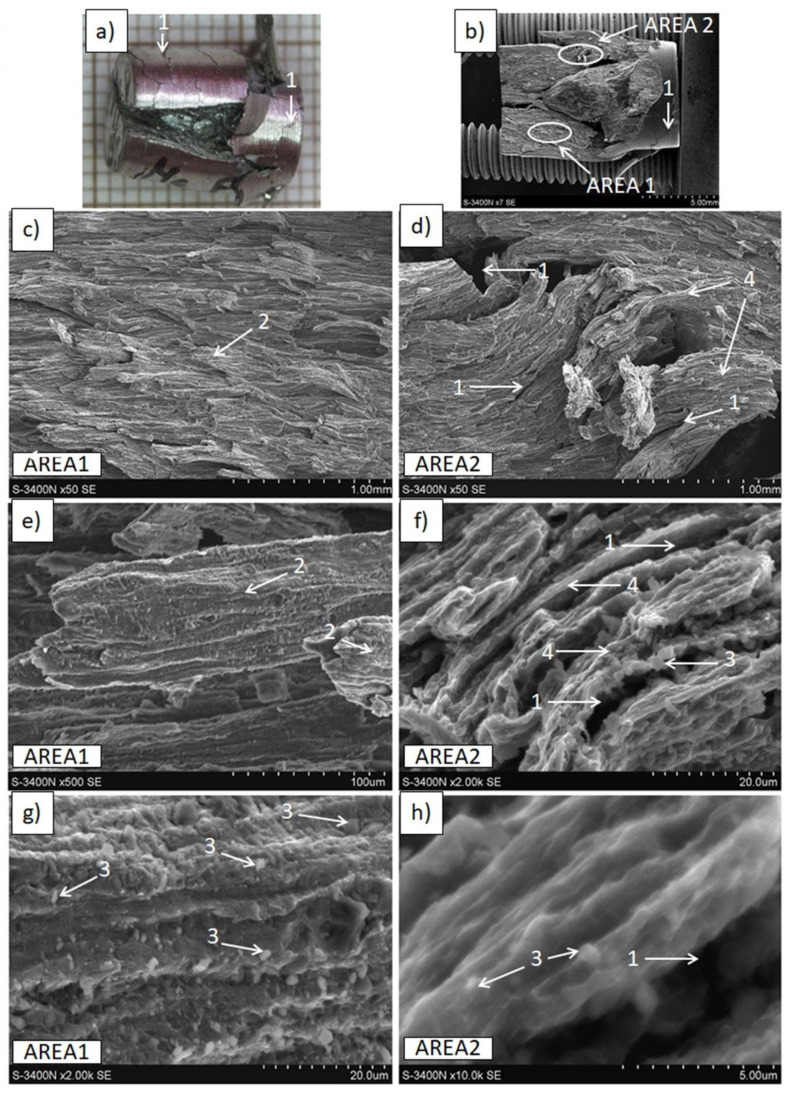
Decohesion effects in the Mg(P) rod after compressive strength test. View of sample (**a**,**b**), fracture of cone and cup type with cracks (1) at the machined surface. SEM micrographs of fracture surface: (**c**,**e**,**g**) splits (2) of deformed fine grained metal with dispersed oxide particles (3), and (**d**,**f**,**h**) metal bands (4) with refined oxides (3) separated by cracks (1).

**Figure 15:**
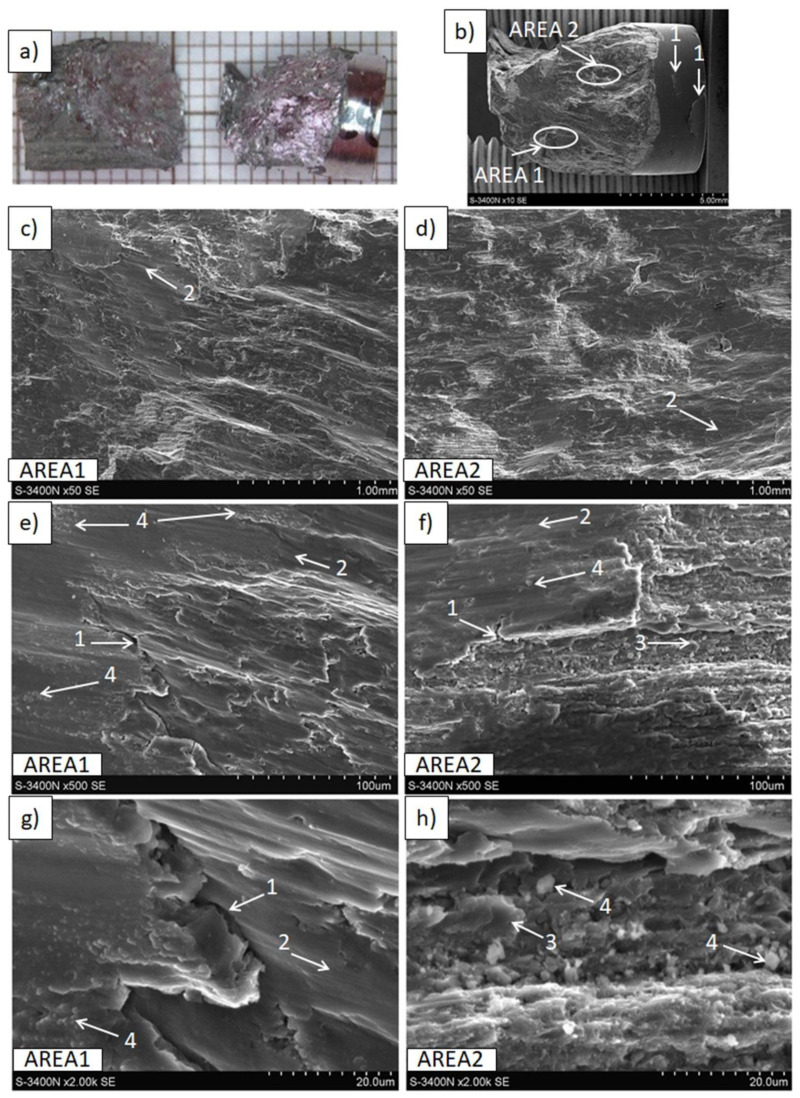
Decohesion effects in the Mg(S) rod after compressive strength test. View of sample (**a**,**b**) fracture of quasi-brittle type with approx. 45° angle and cracks (1) at the machined surface. SEM micrographs of fracture surface: (**c**–**h**) cracks (1), smooth areas of metal (2), areas of refined metal (3), and oxides (4).

**Figure 16:**
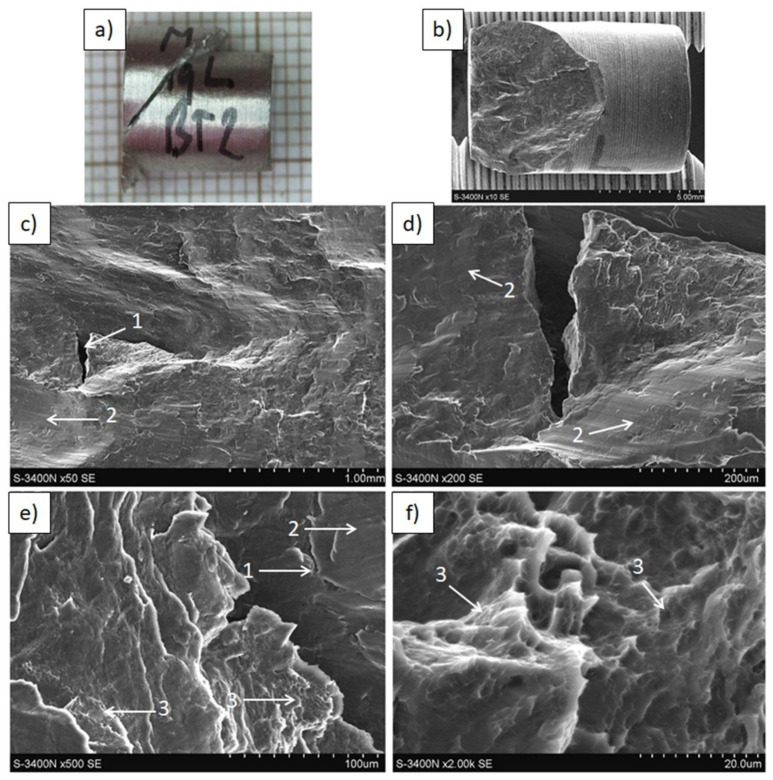
Decohesion effects in Mg(C) rod after compressive strength test. View of sample (**a**,**b**) fracture of quasi-brittle type with approx. 45° angle. SEM micrographs of fracture surface: (**c**–**f**) cracks (1), smooth areas of cleavage metal fracture (2), plastic deformed metal dimples (3).

As opposed to the tensile strength results, the shape of the stress-strain curves for the compressive tests revealed the maximum stress value obtained by both the Mg(P) and Mg(S) rods and their elastic deformation was higher than for Mg(C), particularly for Mg(S) (Figure 10). This effect could have been induced by the presence of MgO and requires further study.

The preliminary results of the application of KOBO extrusion for powdered magnesium processing presented here demonstrated the potential of this SPD method for producing fabrications with a long profile, and revealed some structural aspects that should be considered. Moreover, the performed studies could be used in design parameters for ex situ magnesium composites with micro- and nanosized addition processing, as well as analysis of phenomena occurring in multiphase powdered or compacted systems.

The main directions of further research that can be proposed based on the results obtained herein are:

- The selection of optimum extrusion parameters with respect to the properties of the final products,
- An explanation for the formation of different zones in the cross sections of the processed rods and their influence on the bulk properties,
- characterization of the influence on the magnesium refining processes of the oxide from the magnesium powder surface.

## 4. Conclusions

The experiments presented in this work gave a comparison of the effects of the various forms of magnesium in the KOBO method, when the same main parameters of plastic working, including temperature, extrusion ratio, oscillation frequency, oscillation angle, and extrusion rate were used. The course of extrusion, structural effects, and the properties obtained varied depending on whether cast pure magnesium, cold compacted magnesium powder, or magnesium powder that had been sintered under pressure were applied as the raw materials.

The results can be outlined as follows:

1. The application of magnesium powder in KOBO cold extrusion, both cold compacted and sintered under pressure, gave rods of diameter 8 mm with shiny scales at the surface oriented with the extrusion direction.
2. The sintering of powders under a protective atmosphere prior to the application of the sintered material is recommended for KOBO extrusion. This type of raw material ensures that rods with less porosity, better compaction, higher hardness, and higher tensile and compressive strength parameters are obtained.
3. A comparison of rods fabricated from magnesium powder with respect to those produced from cast magnesium showed an increase in hardness, Young’s modulus, and yield strength, but a decrease in UTS and 0.2% OYS.
4. The applied parameters of KOBO extrusion induced the formation of submicrosized and elongated α-Mg crystallites in powder-based rods. They were similar in size and degree of elongation, but the rods obtained from previously sintered powder clearly had superior properties, due to better material consolidation. The grains in extruded cast material were regular, but less refined, due to more intense dynamic recovery and recrystallization.
5. The presence of oxide film on the surface of the employed powder played a crucial role in the formation of rod microstructure and in the examined decohesion mechanisms. In KOBO extrusion, the MgO film was refined into very fine regular submicro and smaller particles. They were well connected with the strongly refined α-Mg grains and formed bands of oxide-Mg mixture, which were oriented with the extrusion direction but curved in the perpendicular plane, forming a 3D network in the matrix of refined α-Mg crystals.
6. The dynamic recovery and recrystallization of α-Mg deformed by extrusion was limited, because of metal microareas closing in the oxide-α-Mg network, which influenced not only the α-Mg grain size but also the shape and orientation of the boundaries.
7. The oxide–α-Mg network strongly changed the decohesion processes and fracture morphology, because it formed a preferential path for crack propagation. However, in the case of well-compacted material (e.g., Mg(S)), this skeleton caused a significant increase in hardness and elastic properties.

## Figures and Tables

**Table 1:** Time and displacement parameters for maximum extrusion force during KOBO extrusion of magnesium raw materials.

	Maximum Extrusion Force [kN]	Time [s]	Displacement [mm]
Mg(P) 40 × 20 mm	476	42	7.3
Mg(S) 40 × 20 mm	379	30.4	5.2
Mg(C) 40 × 20 mm	624	24	2.7

**Table 2:** Results of open porosity measurements determined by Archimedes method.

POROSITY, %				
Mg(P) after Extrusion	Mg(S) after Sintering	Mg(S) after Extrusion	Mg(C) after Casting	Mg(C) after Extrusion
4.12	5.18	3.92	1.03	0.83

**Table 3:** Lattice parameters, lattice strain, and crystallite size determined for raw materials and rods extruded by KOBO method.

Sample	Lattice Parameters, Å	Lattice Strain, %	Crystallite Size, nm
Mg, reference from ICDD ^1^	a = 3.2095, c = 5.2104	-	-
Mg, powder ^2^	a = 3.211, c = 5.214	0.13 ± 0.01	161 ± 3
Mg(P) rod, perpendicular	a = 3.211, c = 5.212	0.09 ± 0.01	232 ± 4
Mg(P) rod, longitudinal	a = 3.212, c = 5.213	0.09 ± 0.01	271 ± 9
Mg, sintered ^2^	a = 3.210, c = 5.211	0.08 ± 0.01	360 ± 10
Mg(S) rod, perpendicular	a = 3.210, c = 5.211	0.11 ± 0.02	210 ± 10
Mg(S) rod, longitudinal	a = 3.210, c = 5.211	0.11 ± 0.02	270 ± 5
cast Mg (^2^)	a = 3.212, c = 5.212	0.07 ± 0.01	321 ± 8
Mg(C) rod, perpendicular	a = 3.212, c = 5.213	0.14 ± 0.01	297 ± 9
Mg(C) rod, longitudinal	a = 3.211, c = 5.213	0.04 ± 0.01	309 ± 7

^1^—International Center for Diffraction Data, card No. #00-004-0770. ^2^—values for perpendicular and longitudinal directions almost the same.

**Table 4:** Results of tensile and compressive strength tests and HV0.2 measurements determined for KOBO-extruded rods.

Material	Tensile Test			Compressive Test			Hardness HV 0.2		
	UTS	0.2 % OYS	E	σ max	YS	E	Before	Transverse	Longitudinal
	[MPa]	[MPa]	[GPa]	[MPa]	[MPa]	[GPa]	KOBO		
Mg(P)	108.63 ± 7.23	97.48 ± 2.33	30.75 ± 0.92	183 ± 21.5	133.6 ± 3.2	11.1 ± 0.2	34 ± 3.58	46.6 ± 3.4	44.5 ± 4.4
Mg(S)	173.55 ± 1.91	114.5 ± 19.66	33.95 ± 4.03	320.3 ± 10.8	171.6 ± 6.4	12 ± 0.8	33.9 ± 3.7	51 ± 2.9	50.6 ± 3.3
Mg(C)	206.19 ± 2.84	171.23 ± 4.14	22.1 ± 4.1	349 ± 22.4	68.2 ± 0.2	9.4 ± 1.01	32 ± 1.21	34.4 ± 1.4	34.8 ± 3.2

## Data Availability

Not applicable.

## References

[1] Doege E., Dröder K. (2001). Sheet metal forming of magnesium wrought alloys—Formability and process technology. J. Mater. Process. Technol..

[2] Dziubińska A., Gontarz A., Dziedzic K. (2016). Qualitative Research of AZ31 Magnesium Alloy Aircraft Brackets Produced by a New Forging Method. Arch. Metall. Mater..

[3] Śliwa R.E., Balawender T., Hadasik E., Kuc D., Gontarz A., Korbel A., Bochniak W. (2017). Metal Forming of Lightweight Magnesium Alloys for Aviation Applications. Arch. Metall. Mater..

[4] Bednarczyk I., Kuc D., Mikuszewski T. (2018). The Microstructure and Mechanical Properties of Magnesium Alloys Mg-Li-RE after the Process of Casting and Extrusion. Arch. Metall. Mater..

[5] Trzepieciński T., Oleksik V., Pepelnjak T., Najm S.M., Paniti I., Maji K. (2021). Emerging Trends in Single Point Incremental Sheet Forming of Lightweight Metals. Metals.

[6] Luo A.A., Shi R., Miao J. (2021). Review: Magnesium Sheet Alloy Development for Room Temperature Forming. JOM.

[7] Wolff M., Schaper J.G., Suckert M.R., Dahms M., Feyerabend F., Ebel T., Willumeit-Römer R., Klassen T. (2016). Metal Injection Molding (MIM) of Magnesium and Its Alloys. Metals.

[8] Nienaber M., Yi S., Kainer K.U., Letzig D., Bohlen J. (2020). On the Direct Extrusion of Magnesium Wires from Mg-Al-Zn Series Alloys. Metals.

[9] Junak G. (2020). Fatigue Properties of AZ31 and WE43 Magnesium Alloys. Arch. Metall. Mater..

[10] Malaki M., Xu W., Kasar A.K., Menezes P.L., Dieringa H., Varma R.S., Gupta M. (2019). Advanced Metal Matrix Nanocomposites. Metals.

[11] Selva Kumar M., Chandrasekar P., Chandramohan P., Mohanraj M. (2012). Characterisation of titanium–titanium boride composites processed by powder metallurgy techniques. Mater. Charact..

[12] Bian Y., Ni J., Wang C., Zhen J., Hao H., Kong X., Chen H., Li J., Li X., Jia Z. (2021). Microstructure and wear characteristics of in-situ micro/nanoscale niobium carbide reinforced copper composites fabricated through powder metallurgy. Mater. Charact..

[13] Chen J.K., Huang I.S. (2013). Thermal properties of aluminum–graphite composites by powder metallurgy. Compos. Part B Eng..

[14] Abdizadeh H., Ebrahimifard R., Baghchesara M.A. (2014). Investigation of microstructure and mechanical properties of nano MgO reinforced Al composites manufactured by stir casting and powder metallurgy methods: A comparative study. Compos. Part B Eng..

[15] Dutkiewicz J., Kalita D., Maziarz W., Tański T., Borek W., Ostachowski P., Faryna M. (2020). Efect of KOBO extrusion and following cyclic forging on grain refinement of Mg–9Li–2Al–0.5Sc Alloy. Met. Mater. Int..

[16] Bochniak W., Korbel A., Ostachowski P., Ziółkiewicz S., Borowski J. (2013). Extrusion of metals and alloys by KOBO method. Obróbka Plast. Met..

[17] Korbel A., Bochniak W., Borowski J., Błaż L., Ostachowski P., Łagoda M. (2015). Anomalies in precipitation hardening process of 7075 aluminum alloy extruded by KOBO method. J. Mater. Process. Technol..

[18] Dutkiewicz J., Bobrowski P., Rusz S., Hilser O., Tański T., Borek W., Łagoda M., Ostachowski P., Pałka P., Boczkal G. (2018). Efect of various SPD techniques on structure and superplastic deformation of two phase MgLiAl Alloy. Met. Mater. Int..

[19] Korbel A., Bochniak W. (1998). Method of Plastic Forming of Materials. U.S. Patent.

[20] Korbel A., Bochniak W., Ostachowski P., Błaż L. (2011). Visco-plastic flow of metal in dynamic conditions of complex strain scheme. Metall. Mater. Trans. A.

[21] Korbel A., Bochniak W. (2017). Stratified plastic flow in metals. Int. J. Mech. Sci..

[22] Gholinia A., Prangnell P.B., Markushev M.V. (2000). The effect of strain path on the development of deformation structures in Neverly deformed aluminium alloys processed by ECAE. Acta Mater..

[23] Sabirov I., Kolednik O., Valiev R.Z., Pippan R. (2005). Equal channel angular pressing of metal matrix composites: Effect on particle distribution and fracture toughness. Acta Mater..

[24] Pantělejev L., Štěpánek R., Man O. (2015). Thermal stability of bimodal microstructure in magnesium alloy AZ91 processed by ECAP. Mater. Charact..

[25] Tokunaga T., Kaneko K., Horita Z. (2008). Production of aluminum-matrix carbon nanotube composite using high pressure torsion. Mater. Sci. Eng. A.

[26] Al-Zubaydi A., Zhilyae A., Wang S., Reed P. (2015). Superplastic behaviour of AZ91 magnesium alloy processed by high-pressure torsion. Mater. Sci. Eng. A.

[27] Bazarnik P., Romelczyk B., Huang Y., Lewandowska M., Langdon T.G. (2016). Effect of applied pressure on microstructure development and homogeneity in an aluminium Alloy processed by high-pressure torsion. J. Alloys Compd..

[28] Ghalehbandi S.M., Malaki M., Gupta M. (2019). Accumulative Roll Bonding—A Review. Appl. Sci..

[29] Kuśnierz J. (2007). Nanomateriały wytwarzane metodą intensywnych odkształceń plastycznych. Kom. Budowy Masz. PAN—Oddział W Pozn..

[30] Kuśnierz J., Bogucka J. (2005). Accumulative Roll-Bonding (ARB) of Al 99.8%. Arch. Metall. Mater..

[31] Siddesha H.S., Shantharaja M. (2013). Characterization of mechanical properties of aluminum processed by repetitive corrugation and straightening process using taguchi analysis. JOM.

[32] Huang J., Zhu Y.T., Alexander D.J., Liao X., Lowe T.C., Asaro R.J. (2004). Development of repetitive corrugation and straightening. Mater. Sci. Eng. A.

[33] Bhovi P.M., Patil D.C., Kori S.A., Venkateswarlud K., Huange Y., Langdon T.G. (2016). A comparison of repetitive corrugation and straightening and high-pressure torsion using an Al-Mg-Sc Alloy. J. Mater. Res. Technol..

[34] Pustovoytov D., Pesin A., Tandon P. (2021). Asymmetric (Hot, Warm, Cold, Cryo) rolling of light alloys: A Review. Metals.

[35] Biswas S., Kimb D., Suwas S. (2012). Asymmetric and symmetric rolling of magnesium: Evolution of microstructure, texture and mechanical properties. Mater. Sci. Eng. A.

[36] Fajfar P., Lah A.S., Kraner J., Kugler G. (2017). Asymmetric rolling process. Mater. Geoenviron..

[37] Deb S., Panigrahi S.K., Weiss M. (2018). Development of bulk ultrafine grained Al-SiC nano composite sheets by a SPD based hybrid process: Experimental and theoretical studies. Mater. Sci. Eng. A.

[38] Pramono A., Dhoska K., Alhamidi A., Trenggono A., Milandia A. Investigation of mechanical properties on composite materials by several of severe plastic deformation (SPD) methods. Proceedings of the IOP Conference Series: Materials Science and Engineering.

[39] Aristizabal K., Tayrac A., Katzensteiner A., Bachmaier A., Suarez S. (2019). Friction and Tribo-Chemical Behavior of SPD-Processed CNT-Reinforced Composites. Lubricants.

[40] Zwolak M., Śliwa R.E. (2017). Physical modeling of plastic flow in the KOBO extrusion process using dies of different geometry. Obróbka Plast. Met..

[41] Bogucki R., Sulikowska K., Bieda M., Ostachowski P., Sztwiertnia K. (2015). Analysis of microstructure and mechanical properties changes in AA1050 aluminum subjected to ECAP and KoBo processes. Arch. Metall. Mater..

[42] Andrzejewski D., Borowski J., Garbiec D. (2015). Wpływ wyciskania metodą KOBO na wytrzymałość zmęczeniową stopu aluminium 7075. Inżynieria Mater..

[43] Korbel A., Pieła K., Ostachowski P., Łagoda M., Błaż L., Bochniak W., Pawlyta M. (2018). Structural phenomena induced in the course of and post low-temperature KOBO extrusion of AA6013 aluminum alloy. Mater. Sci. Eng. A.

[44] Ziółkiewicz S., Gąsiorkiewicz M., Wesołowska P., Szczepanik S., Szyndler R. (2012). Influence of KOBO treatment on the plastic properties of AM60 magnesium alloy. Obróbka Plast. Met..

[45] Bednarczyk I., Kuc D., Tomaszewska A., Mrugała A. (2017). The influence of extrusion process on the microstructure and mechanical properties of magnesium alloys. Arch. Metall. Mater..

[46] Bednarczyk I. (2018). Characterization of magnesium LAE442 alloy microstructure after deformation process with the use of KoBo method. Inżynieria Mater..

[47] Ziółkiewicz S., Gąsiorkiewicz M., Wesołowska P., Szczepanik S., Szyndler R. (2012). Effect of KOBO treatment on the plastic properties of the AZ31 magnesium alloy. Obróbka Plast. Met..

[48] Bochniak W., Korbel A., Ostachowski P., Łagoda M. (2018). Plastic flow of metals under cyclic change of deformation path conditions. Arch. Civ. Mech. Eng..

[49] Długosz P., Bochniak W., Ostachowski P., Molak R., Guigou M.D., Hebda M. (2021). The influence of conventional or KOBO extrusion process on the properties of AZ91 (MgAl9Zn1) alloy. Materials.

[50] Bochniak W., Ostachowski P., Jagieła S. (2016). Plastic forming of AZ91 alloy using the KOBO method. J. Eng. Mater. Technol..

[51] Rodak K., Brzezińska A., Sobota J. (2020). Refinement of Al-5%Cu and Al-25%Cu alloys by means of KoBo methods. Arch. Metall. Mater..

[52] Rodak K., Kuc D., Mikuszewski T. (2020). Superplastic deformation of Al-Cu alloys after grain refinement by extrusion combined with reversible torsion. Materials.

[53] Brzezińska A., Rodak K. (2018). Refinement of CuCr0.6 alloy by means of KoBo and COT methods. Met. Form..

[54] Korbel A., Bochniak W. (2004). Refinement and control of the metal structure elements by plastic deformation. Scr. Mater..

[55] Ostachowski P., Bochniak W., Łagoda M., Ziółkiewicz S. (2019). Strength properties and structure of CuCrZr alloy subjected to low-temperature KOBO extrusion and heat treatment. Int. J. Adv. Manuf. Technol..

[56] Woźniak J., Kostecki M., Bochniak W., Olszyna A. (2012). Al/SiC composites produced by direct extrusion using the KOBO method. Inżynieria Mater..

[57] Olszówka-Myalska A., Kuc D., Myalski J., Chrapoński J. (2019). Effect of Magnesium Matrix Grain Refinement Induced by Plastic Deformation in a Composite with Short Carbon Fibers. Metals.

[58] Wieczorek J., Oleksiak B., Łabaj J., Węcki B., Mańka M. (2016). Silver matrix composites—Structure and properties. Arch. Metall. Mater..

[59] Olszówka-Myalska A., Wrześniowski P., Myalska H., Godzierz M., Kuc D. (2019). Impact of the Morphology of Micro- and Nanosized Powder Mixtures on the Microstructure of Mg-Mg_2_Si-CNT Composite Sinters. Materials.

[60] Korbel W., Bochniak P., Ostachowski A., Paliborek M., Łagoda A., Brzostowicz A. (2016). new constitutive approach to large strain plastic deformation. Int. J. Mater. Res..

[61] Rietveld H.M. (1967). Line profiles of neutron powder-diffraction peaks for structure refinement. Acta Crystallogr..

[62] Rietveld H.M. (1969). A profile refinement method for nuclear and magnetic structures, Journal of Applied Crystallography. J. Appl. Crystallogr..

[63] Toby B.H. (2006). R factors in Rietveld analysis: How good is good enough?. Powder Diffr..

[64] Karolus M., Łagiewka E. (2004). Crystallite size and lattice strain in nanocrystalline Ni-Mo alloys studied by Rietveld refinement. J. Alloys Compd..

[65] Wang Q.J., Du Z.Z., Luo L., Wang W. (2012). Fatigue properties of ultra-fine grain Cu–Cr alloy processed by equal-channel angular pressing. J. Alloy. Compd..

[66] Stolyarov V.V., Lapovok R., Brodova I.G., Thomson P.F. (2003). Ultrafine-grained Al-/5 wt.% Fe alloy processed by ECAP with back pressure. Mater. Sci. Eng. A.

[67] Segal V.M. (2005). Deformation mode and plastic flow in ultra fine grained metals. Mater. Sci. Eng. A.

[68] Jia H., Bjørge R., Marthinsen K., Mathiesen R.H., Li Y. (2017). Soft particles assisted grain refinement and strengthening of an Al-Bi-Zn alloy subjected to ECAP. Mater. Sci. Eng. A.

